# Functional and Radiological Outcomes of Allograft Application in Distal Femur Fractures: A Retrospective Study

**DOI:** 10.7759/cureus.82473

**Published:** 2025-04-17

**Authors:** Ashwin Kumar, Sagar Venkataraman, Nagakumar J S, Gils Thampi

**Affiliations:** 1 Department of Orthopedics, Sri Devaraj Urs Medical College (SDUMC), Kolar, IND

**Keywords:** allograft, bone healing, distal femur fractures, functional outcomes, radiological union

## Abstract

Background

Distal femur fractures are complex injuries requiring surgical intervention, particularly in cases involving bone loss, comminution, or osteoporosis. While autografts remain the preferred option for bone grafting due to their osteogenic, osteoinductive, and osteoconductive properties, their use is often restricted due to donor-site morbidity, limited availability, and increased surgical time. Allografts provide an alternative, offering structural support without additional surgical site morbidity. This study evaluates the radiological and functional outcomes of allograft application in distal femur fractures.

Methods

A retrospective study was conducted at R. L. Jalappa Hospital, Kolar, analyzing 23 patients who underwent allograft-assisted distal femur fracture fixation between May 2023 and April 2024. The inclusion criteria were distal femur fractures requiring allograft augmentation, while exclusion criteria included pathological fractures, severe infections, and polytrauma patients. Radiological union was assessed using serial X-rays at three and six months, evaluating callus formation and cortical bridging. Pain relief was measured using the visual analog scale (VAS) at each follow-up. Functional recovery was evaluated using the Western Ontario and McMaster Universities Osteoarthritis Index (WOMAC score), assessing pain, stiffness, and physical function.

Results

Radiological signs of union were achieved in most patients within three to six months. By three months, radiological healing was observed in 11 (47.8%) of cases, and by six months, 17 (73.9%) of patients achieved cortical union. Pain score improved significantly over time, with VAS scores decreasing from 6-8 at one month to 1-3 at six months. Functional recovery followed a similar pattern, with WOMAC scores improving from 65-80 at one month to 85-98 at six months, indicating substantial progress in joint mobility, weight-bearing ability, and pain reduction.

Conclusion

The findings suggest that allografts provide effective structural support and facilitate bone healing in distal femur fractures, particularly in cases with bone loss. Radiological union is slightly slower than the autografts, and the functional outcomes remain comparable. Allografts serve as a viable option for patients with large bone defects or contraindications for autograft harvesting.

## Introduction

Distal femur fractures are complex injuries that pose significant challenges in terms of surgical management and functional recovery. Distal femur fractures represent 6% of all femoral fractures and are often caused by high-energy trauma in younger individuals and low-energy falls in elderly patients with osteoporosis [1]. Due to their anatomical location near the knee joint, these fractures are associated with a high risk of complications, including malunion, nonunion, post-traumatic arthritis, and implant failure [2,3]. The management of these fractures remains challenging due to factors such as comminution, bone loss, and the need for anatomical realignment to restore limb function. The choice of surgical technique and implant plays a crucial role in ensuring optimal outcomes, with stable fixation and early mobilization being key objectives [2].

In cases with bone loss or poor healing potential, bone grafting is required to promote osteogenesis, osteoinduction, and osteoconduction [4]. Autografts, commonly obtained from the iliac crest, fibula, or proximal tibia, provide superior osteogenic properties but are associated with donor-site pain, hematoma formation, and infection risks. In cases with severe bone loss or comminution, structural bone grafts are often required to support fracture healing and maintain limb stability. While autografts remain the gold standard due to their osteogenic, osteoinductive, and osteoconductive properties, they are associated with donor-site morbidity, including pain and complications related to bone harvesting [5,6]. Allografts, on the other hand, eliminate donor-site morbidity while offering structural stability for fracture healing. As an alternative, allografts have gained popularity for their ability to provide structural support while eliminating donor-site complications [7]. These grafts act as a biological scaffold, promoting bone remodeling and regeneration in cases of extensive bone loss [4]. 

Despite the increasing use of allografts in orthopedic trauma, their impact on functional and radiological outcomes in distal femur fractures remains an area of ongoing research. This retrospective study aims to evaluate the role of allografts in the management of distal femur fractures by assessing postoperative radiological parameters and functional recovery. The findings may contribute to refining treatment strategies and optimizing clinical outcomes in patients undergoing surgical fixation with allograft augmentation [2,4,6].

## Materials and methods

Study design 

A retrospective study was conducted at R. L. Jalappa Hospital, Kolar, between May 2023 and April 2024. 

Study population and sample size

Patients who underwent allograft application for distal femur fractures were included in the study. The inclusion criteria were distal femur fractures requiring allograft augmentation, while exclusion criteria included pathological fractures, severe infections, and polytrauma patients. Data retrieved from the patient's medical records were thoroughly analyzed with at least a six-month follow-up. A total of 23 patients who met the inclusion and exclusion criteria were analyzed in this study.

Study measures

The aim of the study was to assess radiological union times in allograft-assisted distal femur fractures, evaluate pain relief using the visual analog scale (VAS) scores over a six-month follow-up, and analyze functional outcomes using the Western Ontario and McMaster Universities Osteoarthritis Index (WOMAC score) to determine mobility, stiffness, and pain improvement.

Ethics statement

Ethical clearance was received from the Institutional Ethical Committee of Sri Devaraj Urs Medical College (approval number SDUMC/KLR/R&D/CEC/S/PG/124/2024-25) to conduct the research. 

Statistical analysis

Data will be entered in MS Excel (Microsoft Corporation, Redmond, Washington, United States) and analyzed using IBM SPSS Statistics for Windows, Version 25 (Released 2017; IBM Corp., Armonk, New York, United States). Continuous variables are presented as mean, standard deviation (SD), median, minimum, and maximum. Categorical variables are presented as frequency and percentage.

## Results

In our study, the total number of participants was 23. Table 1 presents the demographic characteristics of the study population. The mean age of participants was 41.35 years (SD = 12.2). The sample consisted of 10 (43.5%) males and 13 (56.5%) females.

**Table 1:** Descriptive statistics of the study population (n = 23)

Variable	n	%
Gender		
Male	10	43.5%
Female	13	56.5%
Age (years)	41.35 (12.2)	

Radiological assessment showed a progressive increase in bone healing over the follow-up period. By three months, 11 (47.8%) patients demonstrated radiological signs of healing, characterized by partial callus formation and cortical bridging. At six months, 17 (73.9%) patients achieved full radiological union, indicating successful bone healing with allograft augmentation. Four (17.3%) had signs of union after six months but didn't achieve complete cortical union (Table 2).

**Table 2:** Radiological union over time (n = 23)

Time point	No union (n, %)	Union achieved (n, %)
3 months	12 (52.2%)	11 (47.8%)
6 months	6 (26.08%)	17 (73.9%)

Pain levels decreased significantly over time, as measured by the VAS. The mean VAS score at one month was 7.13, indicating moderate to severe pain. By three months, the mean score had reduced to 5.61, reflecting partial pain relief. At six months, the VAS score further improved to 4.30, signifying a substantial reduction in pain and improved patient comfort (Table 3).

**Table 3:** VAS score of study participants over time (n = 23) VAS: visual analog scale

Time point	Mean	SD	Min	Max
1 month	7.13	0.63	6.0	8.0
3 months	5.61	0.89	4.0	7.0
6 months	4.30	0.97	2.0	6.0

Functional recovery, assessed using the WOMAC score, showed a steady improvement throughout the study period. At one month, the mean WOMAC score was 71.3, indicating moderate impairment in joint function, stiffness, and pain. By three months, the score improved to 78.7, reflecting better mobility and reduced stiffness. At six months, the mean WOMAC score reached 85.6, demonstrating significant functional recovery, enhanced joint mobility, and improved weight-bearing ability (Table 4).

**Table 4:** WOMAC scores over time (n = 23) WOMAC score: Western Ontario and McMaster Universities Osteoarthritis Index

Time point	Mean	SD	Min	Max
1 month	71.3	4.08	65.0	79.0
3 months	78.7	3.84	71.0	86.0
6 months	85.6	3.68	78.0	93.0

In our study, five (21.7%) patients developed complications such as infection and nonunion. Out of which, three (13.0%) patients developed infection, and two (8.7%) patients had no signs of union after six months (Table 5).

**Table 5:** Complications (n = 23)

Complications	n	%
Infection	3	13.0
Nonunion	2	8.7

## Discussion

In this study, radiological signs of union were achieved in most patients within three to six months following allograft-assisted fixation of distal femur fractures. At three months, radiological signs of healing were observed in 11 (47.8%) patients. This early phase likely reflects the initial biological process of callus formation, which is essential for subsequent cortical bridging. By six months, 17 (73.9%) patients achieved full cortical union.

These findings are in agreement with previous studies on distal femur fracture management. Ehlinger et al. [2] noted that a gradual progression in fracture healing is typical, with radiological signs of union emerging between three and six months postoperatively. Another study emphasized that allografts provide an effective structural scaffold for bone regeneration; however, the incorporation process via creeping substitution is inherently slower than that of autografts [4]. This difference in biological integration likely accounts for the delayed early union observed in our study. Furthermore, while autografts remain the gold standard due to their osteogenic, osteoinductive, and osteoconductive properties, their use is often limited by donor-site morbidity and availability issues [5,6]. 

Comparative healing patterns: allografts vs. autografts

In this study, comparative healing patterns of autografts and allografts showed autografts achieving faster bone integration due to their osteogenic properties, typically showing union within 8-12 weeks. In contrast, allografts require 12-16 weeks, as they lack viable osteogenic cells, aligning with our study findings of radiological union at 3-6 months [8,9]. Several reports confirm similar healing timelines for allografts in long bone fractures [10,11].

In our study, the significant decline in pain levels, as reflected by the decrease in VAS scores from 6-8 at one month to 1-3 at six months, underscores the effectiveness of allograft-assisted fixation in alleviating postoperative discomfort. This reduction in pain is likely attributable to improved stability and progressive fracture healing that facilitate early mobilization. In parallel, the enhancement in functional outcomes, with WOMAC scores rising from 65-80 at one month to 85-98 at six months, suggests substantial recovery in joint mobility, reduced stiffness, and improved weight-bearing capacity. These findings are in line with previous studies; for instance, Ehlinger et al. observed similar improvements in pain and functional parameters following distal femur fracture management, highlighting the role of effective structural support in postoperative recovery [2]. In our study, five (21.7%) patients developed complications such as infection and nonunion. Out of which, three (13.0%) patients developed infection, and two (8.7%) patients had no signs of union after six months.

These results also underscore the clinical relevance of allografts as a viable alternative to autografts, particularly in patients where donor-site morbidity is a concern. Studies by Laurie et al. and Arrington et al. have documented the complications associated with autograft harvesting, thereby supporting the shift toward allograft usage in appropriate clinical scenarios [5,6]. The steady improvement in WOMAC scores further reinforces that, as the structural integrity of the fracture site is restored through allograft incorporation, patients experience a commensurate enhancement in function and overall quality of life. In summary, the observed trends in pain reduction and functional recovery align with the broader literature, confirming that allograft-assisted fixation not only facilitates radiological union but also substantially improves patient-reported outcomes in the management of distal femur fractures. Pain reduction was consistent with previous studies, showing VAS score improvement over six months [12]. WOMAC score improvements highlight the role of early physiotherapy and rehabilitation in restoring joint function [13,14].

Limitations

Despite these encouraging outcomes, the study has several limitations. The retrospective design and relatively small sample size may limit the generalizability of the findings. Additionally, the absence of a control group restricts the ability to directly compare allograft outcomes with alternative treatment options. Despite their advantages, allografts pose risks, including delayed union, graft rejection, and potential infection risks [15]. Modern sterilization techniques have minimized immunogenic responses, making allografts a safer alternative for clinical use [16]. The follow-up period of six months, while adequate to capture early healing and functional improvements, may not fully reflect long-term outcomes or potential late complications. However, further randomized controlled trials are needed to assess long-term functional outcomes and implant longevity [17,5].

## Conclusions

The study demonstrates that allograft-assisted fixation of distal femur fractures leads to progressive radiological union over a six-month period. Patients achieved signs of union at three months; however, full cortical union was evident in 17 (73.9%) patients by six months. This structural consolidation was accompanied by a significant reduction in pain, as indicated by a decrease in mean VAS scores from 7.13 at one month to 4.30 at six months. Furthermore, functional recovery improved steadily, with WOMAC scores rising from 71.3 at one month to 85.6 at six months, reflecting enhanced joint mobility, reduced stiffness, and improved weight-bearing ability.
